# Genetic variants and social benefit receipt in premenopausal women with breast cancer treated with docetaxel: a Danish population-based cohort study

**DOI:** 10.1007/s10549-024-07474-9

**Published:** 2024-09-20

**Authors:** Julie A. Schmidt, Cathrine F. Hjorth, Dóra K. Farkas, Per Damkier, Søren Feddersen, Stephen Hamilton-Dutoit, Bent Ejlertsen, Timothy L. Lash, Thomas P. Ahern, Deirdre Cronin-Fenton

**Affiliations:** 1 Department of Clinical Epidemiology, Department of Clinical Medicine, Aarhus University and Aarhus University Hospital, Olof Palmes Allé 43-45, 8200 Aarhus N, Denmark; 2 Department of Clinical Pharmacology, Odense University Hospital, Odense, Denmark; 3 Department of Clinical Research, University of Southern Denmark, Odense, Denmark; 4 Department of Clinical Biochemistry, Odense University Hospital, Odense, Denmark; 5 Department of Clinical Medicine and Department of Pathology, Aarhus University Hospital, Aarhus, Denmark; 6 Danish Breast Cancer Group, Department of Oncology, Rigshospitalet, University of Copenhagen, Copenhagen, Denmark; 7 Department of Clinical Medicine, Faculty of Health and Medical Sciences, University of Copenhagen, Copenhagen, Denmark; 8 Department of Epidemiology, Rollins School of Public Health, Emory University, Atlanta, GA USA; 9 Department of Surgery, The Robert Larner, M.D. College of Medicine, The University of Vermont, Burlington, VT USA

**Keywords:** Breast cancer, Single-nucleotide polymorphisms, Social benefits, Survivorship, Taxane

## Abstract

**Purpose:**

Breast cancer patients’ need for social benefits may increase following taxane-based chemotherapy, due to long-lasting side effects. Specific single nucleotide polymorphisms (SNPs) may mediate such side effects. We investigated the association between SNPs related to taxane metabolism, transport, toxicity, or DNA and neural repair, and receipt of social benefits.

**Methods:**

From the Danish Breast Cancer Group, we identified premenopausal women diagnosed with stage I–III breast cancer during 2007–2011 and treated with docetaxel-based chemotherapy. We genotyped 21 SNPs from archived breast tumors using TaqMan assays. We ascertained social benefit payments from 1 year before to 5 years after diagnosis, using nationwide, administrative registry data. For each week, we categorized women as receiving health-related benefits (including sick leave and disability pension), labor market-related benefits (including unemployment benefits), or as being self-supporting. We computed rate ratios (RRs) of social benefit receipt for variant carriers (heterozygotes plus homozygotes) vs. non-carriers, using negative binominal regression with robust variance estimation.

**Results:**

Among 2430 women, 12% received health-related benefits before diagnosis, 80% at diagnosis, and ~ 24% 2 to 5 years after diagnosis. Labor market-related benefits were uncommon (3–6%). All RRs were near-null and/or imprecise.

**Conclusion:**

We found no clinically meaningful impact of the selected SNPs on social benefit receipt among premenopausal breast cancer survivors treated with docetaxel.

**Supplementary Information:**

The online version contains supplementary material available at 10.1007/s10549-024-07474-9.

## Introduction

Women aged less than 55 years represent over 40% of newly diagnosed breast cancer cases [1, 2]. High incidence rates and improved survival have increased the number of breast cancer survivors, many of whom are working at the time of diagnosis.

Taxane-based adjuvant chemotherapy (docetaxel or paclitaxel) is a cornerstone of breast cancer treatment, yet may be associated with potentially long-lasting, severe, and even debilitating side effects including peripheral neuropathy and fatigue [3–5]. These side effects may compromise recovery after breast cancer. Women treated with chemotherapy are more likely to experience sick leave [6, 7] and impaired return-to-work [8] compared with those not receiving chemotherapy. Loss of work among cancer survivors has a negative impact on quality of life [8] and may have adverse consequences for the economy of both the individual patient and society [8, 9].

The severity of peripheral neuropathy after taxane-based chemotherapy may partly depend on single nucleotide polymorphisms (SNPs) in genes encoding enzymes involved in taxane metabolism, transport, toxicity, and in DNA or neural repair [10–14]. In turn, these genetic variants may help identify patients in greater need for sick leave compensation, disability pension, or other benefits. In our recent study conducted within the Predictors of Breast Cancer Recurrence (ProBe CaRe) cohort, we investigated the association between selected SNPs and return-to-work after a breast cancer diagnosis [15]. The study showed that most women returned to work within 1 year of diagnosis. Our findings also suggested that women homozygous for the *CYP3A5* rs776746 allele took longer to return-to-work compared with non-carriers, although the estimate of association was imprecise. The association between SNPs and receipt of specific types of social benefits has not been examined.

We identified 21 SNPs related to taxane metabolism, transport, toxicity, and DNA and neural repair. We investigated their association with receipt of social benefits, from 1 year before diagnosis to 5 years after diagnosis, among premenopausal women diagnosed with breast cancer.

## Materials and methods

### Setting and design

We conducted a population-based cohort study in Denmark, linking individual-level data from nationwide registries, clinical databases, and biobanks. Data linkage was accomplished with the civil personal registration number, a unique identifier assigned to all residents of Denmark at birth or immigration [16].

Denmark has a tax-supported healthcare system, ensuring free-of-charge care provided to all residents by general practitioners in the community and by hospitals [16]. Patients with invasive breast cancer are registered in the Danish Breast Cancer Group (DBCG) clinical database, which compiles clinical and demographic characteristics at the time of breast cancer diagnosis including tumor stage, grade, human epidermal growth factor receptor 2 (HER2), estrogen receptor (ER) status, guideline-based recommended treatments (including chemotherapy), received treatments, age, and menopausal status [17, 18]. In Denmark, all diagnostic formalin-fixed paraffin-embedded (FFPE) tissue biopsies are routinely archived in perpetuity at local pathology departments, and logged in the Danish National Pathology Registry [19].

In addition to tax-supported healthcare, the Danish government offers subsistence and unemployment payments, student grants, and a range of other social and health-related benefits to residents in need. Such transfer payments have been registered on a weekly basis in the Danish Register for Evaluation of Marginalization (DREAM) since 1991 [20, 21].

Information on comorbidities, cohabitation, and level of education were obtained from the Danish National Patient Registry [22], Statistics Denmark [23], and the Danish Population’s Education Registry [24], respectively.

### Study cohort

The ProBe CaRe cohort includes 5959 premenopausal women diagnosed with stage I–III breast cancer across Denmark from 2002 to 2011 and registered in the DBCG [25, 26]. Only women with ER-positive tumors who received tamoxifen (ER +/TAM +) and women with ER-negative tumors who did not receive tamoxifen (ER −/TAM −) were eligible for cohort entry. In 2007, docetaxel was designated as guideline chemotherapy (in combination with cyclophosphamide and, for most, epirubicin [27, 28]). Thus, we restricted the study population to women diagnosed from 2007 onwards for whom chemotherapy was recommended [28] (﻿Supplementary file1, Supplemental Fig. 1). We included women aged 18–55 years at diagnosis to ensure women were of working age throughout the study period. Finally, we restricted our sample to women with available FFPE tumor blocks who resided in Denmark during the year preceding diagnosis.

The study was approved by the Danish Data Protection Agency (AU 2016-051-000001, #808), the Regional Ethics Committee (record no. 1-10-72-4-18), and the DBCG (DBCG-2018-01-04).

### Genetic variants in breast tumor tissue

We investigated 21 candidate SNPs in 16 genes coding for enzymes involved in taxane metabolism (*CYP1B1, CYP3A, CYP3A4, CYP3A5,* and *GSTP1*), taxane transport (*ABCB1, ABCC2, ABCG2, SLCO1B1,* and *SLCO1B3)*, DNA repair (*ERCC1* and *ERCC2),* and taxane-related toxicity or neural repair (*EPHA4, EPHA5, EPHA6,* and *FGD4*) [14, 15, 29]. Archived breast tumor tissue blocks were collected for eligible ProBe CaRe participants. DNA was extracted and genotyped for the selected SNPs using commercially available TaqMan assays on a StepOne Plus real-time instrument (Applied Biosystems, Thermo Fischer Scientific, Foster City, CA, USA), as previously described [14, 15, 29]. RefSeq numbers (rs) and assay details of the SNPs are provided in Supplementary file1, Supplementary Table 1.

We categorized women based on number of variant alleles as non-carriers (carrying no variant allele on either locus) or carriers (carrying the variant on one locus [heterozygotes] or on both loci [homozygotes]) for each gene.

### Social benefit use

We used the DREAM database, which contains weekly records of all transfer payments, to access information on receipt of social benefits. We collected information from 1 year before to 5 years after the breast cancer diagnosis date (i.e., date of breast cancer surgery). Given the diagnostic period of our study population (2007–2011), we examined data on social benefits from 2006–2016.

Specifically for sick leave, only ‘long-term’ leave is captured by DREAM [21, 30]. Conversely, ‘short-term’ sick leave is not registered. ‘Short-term’ is defined as the initial period in which the employer must cover the cost of sick leave compensation, ranging from 14 to 30 days during our study period [31]. Absence lasting longer than this period is defined as ‘long-term’. After the initial period, the employer is reimbursed by the municipality for the sick leave compensation, which is registered in DREAM [21, 30]. In addition, the initial period is also registered, and DREAM thus reflects the entire length of ‘long-term’ sick leave (from the first day of absence).

For each week, we categorized women into one of three social benefit categories: receiving health-related benefits, receiving labor market-related benefits, and self-supporting or receiving student grants (referred to as ‘self-supporting’; Table 1). We categorized women who received benefits due to maternity leave according to their social benefit category during the week immediately before the start of maternity leave. If women had retired, emigrated, or died they were placed into a fourth censored category. Full details of DREAM codes used for categorizing cohort members are provided in Supplementary file1, Supplementary Table 2.

**Table 1:** Social benefit categories classified using the DREAM database

Category	Types of benefits
Health-related benefits	‘Long-term’ sick leave^a^
	Disability pension
	Flexi job scheme (helping individuals with reduced ability to work and thus stay in the labor market [32])
	Early retirement after flexi job
	Vocational rehabilitation programs and determination of work ability (often used before granting a disability pension)
Labor market-related benefits	Unemployment, including part-time unemployment
	Social security/assistance
Self-supporting	No benefit as proxy for employed or self-supporting
	Student grants and adult traineeships

^a^‘Short-term’ sick leave is paid by the employer and thus not captured by DREAM. During our study period, ‘short-term’ sick leave ranged from 14 to 30 days [31]

### Covariates

Because CYP family genes are also involved in tamoxifen metabolism [14], associations between SNPs and social benefit receipt could potentially be affected by ER-status and receipt of tamoxifen. We therefore used the DBCG database to ascertain data on ER-status and receipt of tamoxifen. Other covariates considered were age at diagnosis, additional clinical and treatment characteristics (including tumor stage, grade, HER2, type of surgery, and radiotherapy [17, 18]), comorbidities categorized using the Charlson Comorbidity Index (CCI) Score [33], cohabitation status (cohabiting; living alone), and the highest attained educational level at diagnosis (short [~ 9–11 years of schooling]; intermediate [~ 12–13 years of schooling]; long [~ 14–20 years of schooling]).

### Statistical analysis

Participant characteristics were described with frequencies and proportions.

We visually described receipt of social benefits by plotting the proportion of women in each of the social benefit categories in weekly intervals.

We estimated the association of SNPs with the number of weeks spent in each of the three social benefit categories (i.e., receiving health-related benefits, receiving labor market-related benefits, and self-supporting) and the associated rate ratio (RR), 95% confidence intervals (CI) and p-values using negative binominal regression models with a robust variance estimator. Differences in log counts between variant carriers and non-carriers can be interpreted as log RR when follow-up is constant. To account for differences in social benefit receipt by time period with respect to breast cancer diagnosis, we stratified all analyses by time (− 12–0, 1–6, 7–12, 13–24, and 25–60 months from the diagnosis date). We used unadjusted models because variant alleles are allocated randomly at conception and thus causally unrelated to covariates. We used volcano plots (− log(p) against RR) to visually summarize the results. To represent the uncertainty of the RR, we plotted the ratio between the upper and lower limit of the 95% CI (UL/LL) against the RR; a smaller ratio represents a narrower CI.

In exploratory analyses, we ran the same models separately for heterozygotes and homozygotes compared with non-carriers. Because of the low minor allele frequency of some SNPs and resulting low statistical power, we only report results for SNPs with at least 100 homozygous variant women. We also repeated the main analyses in women in the ER +/TAM + group.

All analyses were conducted using SAS software version 9.4 (SAS Institute Inc., Cary, NC, USA).

## Results

The study population included 2430 women after exclusions (Supplementary file1, Supplementary Fig. 1). Most women were diagnosed at ages 45–55 years (56%), with tumors that were ER + (78%), HER2 negative (75%), stage II (56%), and grade 2 (42%) or 3 (33%) (Table 2). Treatment most often included lumpectomy (61%), and all women with ER + tumors received tamoxifen. Most had a comorbidity score of zero (87%), were living with a partner (76%), and had completed an intermediate (48%) or long (34%) education.

**Table 2:** Characteristics of 2430 premenopausal women with breast cancer who were intended to receive treatment with docetaxel in Denmark (2007–2011)

Characteristics	N	%^a^
Total	2430	100
Age at diagnosis, years		
18–34	172	7
35–44	901	37
45–55	1357	56
ER-status and endocrine therapy		
ER–/TAM–	535	22
ER +/TAM +	1895	78
HER2 status		
Negative	1820	75
Positive	447	18
Unknown	163	7
Stage		
Stage I	619	26
Stage II	1367	56
Stage III	426	18
Missing	18	1
Histological grade		
Grade 1	364	15
Grade 2	1010	42
Grade 3	798	33
Not graded	229	9
Missing	29	1
Surgery and radiotherapy		
Mastectomy without intended radiotherapy	327	14
Mastectomy and intended radiotherapy	615	25
Lumpectomy	1488	61
Comorbidity, CCI score		
0	2108	87
1	165	7
2	70	3
3 +	87	4
Cohabitation		
Cohabiting	1857	76
Living alone	566	23
Unknown	7	0
Education level		
Short (~ 9–11 years of schooling)	412	17
Intermediate (~ 12–13 years of schooling)	1164	48
Long (~ 14–20 years of schooling)	826	34
Unknown	28	1

^a^Percentages may not add to 100% because of rounding

*CCI* Charlson Comorbidity Index [33], *ER* estrogen receptor, *ER +/TAM +* women with ER-positive tumors who received tamoxifen, *ER −/TAM −* women with ER-negative tumors who did not receive tamoxifen, *HER2* human epidermal growth factor receptor 2

### Receipt of social benefits

**Fig. 1:**
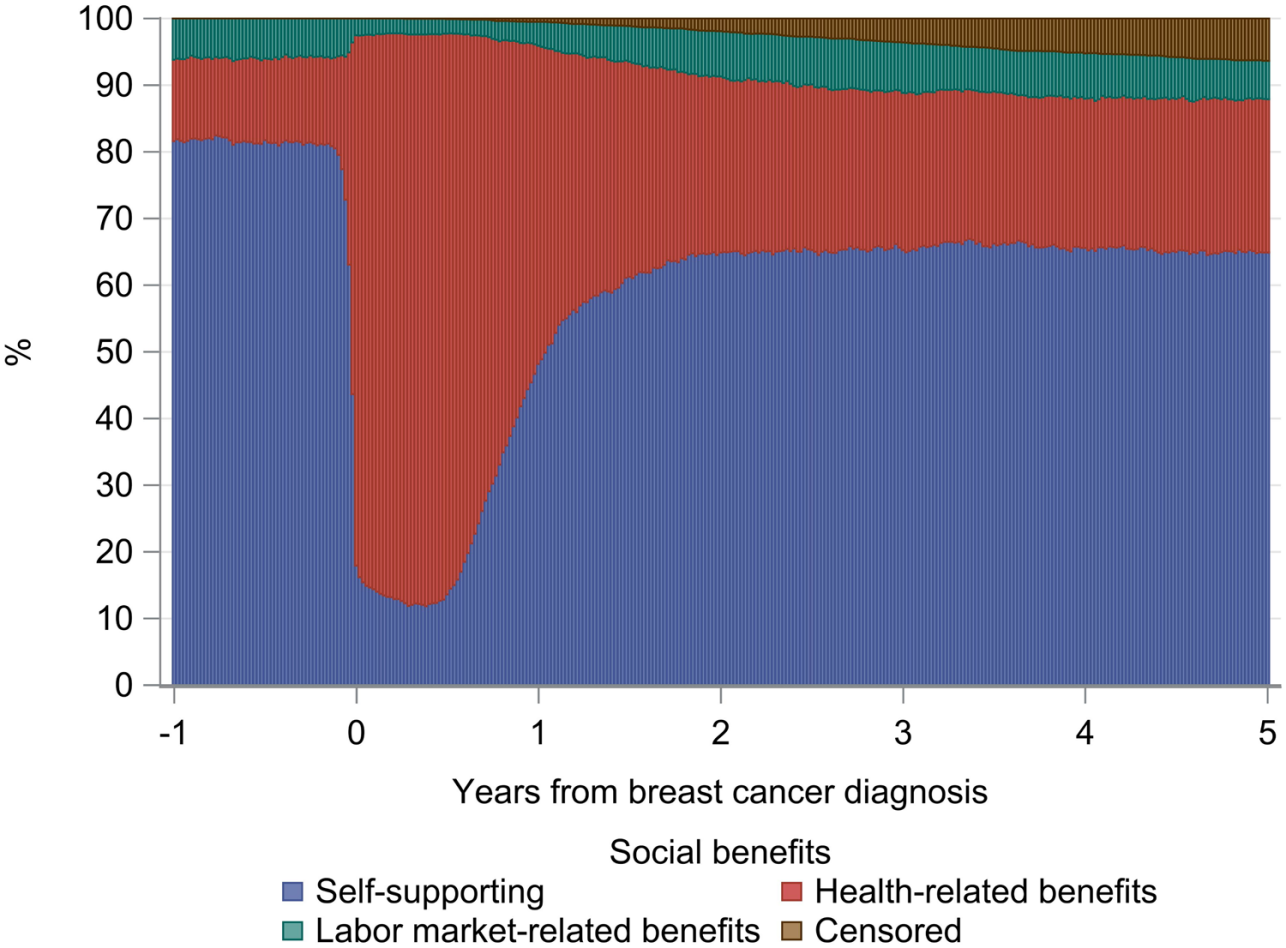
Percent of women receiving social benefits from 1 year prior to 5 years after breast cancer diagnosis among 2430 premenopausal women with breast cancer in Denmark (2007–2011). Details of the categorization are available in Table 1 and in ﻿Supplementary file1, Supplementary Table 2

Figure 1 shows social benefit receipt, from 1 year before to 5 years after breast cancer diagnosis, in the entire study population regardless of genetic variants. Percentages of women in each social benefit category 1 year before diagnosis, at diagnosis, and at the end of the 6th, 12th, 24th, and 60th month after diagnosis are shown in ﻿Supplementary file2, Supplementary Table 3. One year before diagnosis, 12% received health-related benefits and 6% received labor market-related benefits, while 82% were self-supporting. As expected, the proportion of women receiving health-related benefits increased markedly from a few weeks prior to diagnosis to the diagnosis date, at which point 80% of women received health-related benefits, 3% received labor market-related benefits, and 18% were self-supporting. This pattern was observed until six months after diagnosis, the period during which patients typically undergo surgery, chemotherapy, and radiotherapy. From 6 to 24 months after diagnosis, receipt of social benefits fell, driven by fewer women receiving health-related benefits (26% at the end of the 24th month). From then onwards, the pattern stabilized with approximately 23% receiving health-related benefits. In the same period, 6% received labor market-related social benefits and 65% were self-supporting.

### Genetic variants and receipt of social benefits

**Fig. 2:**
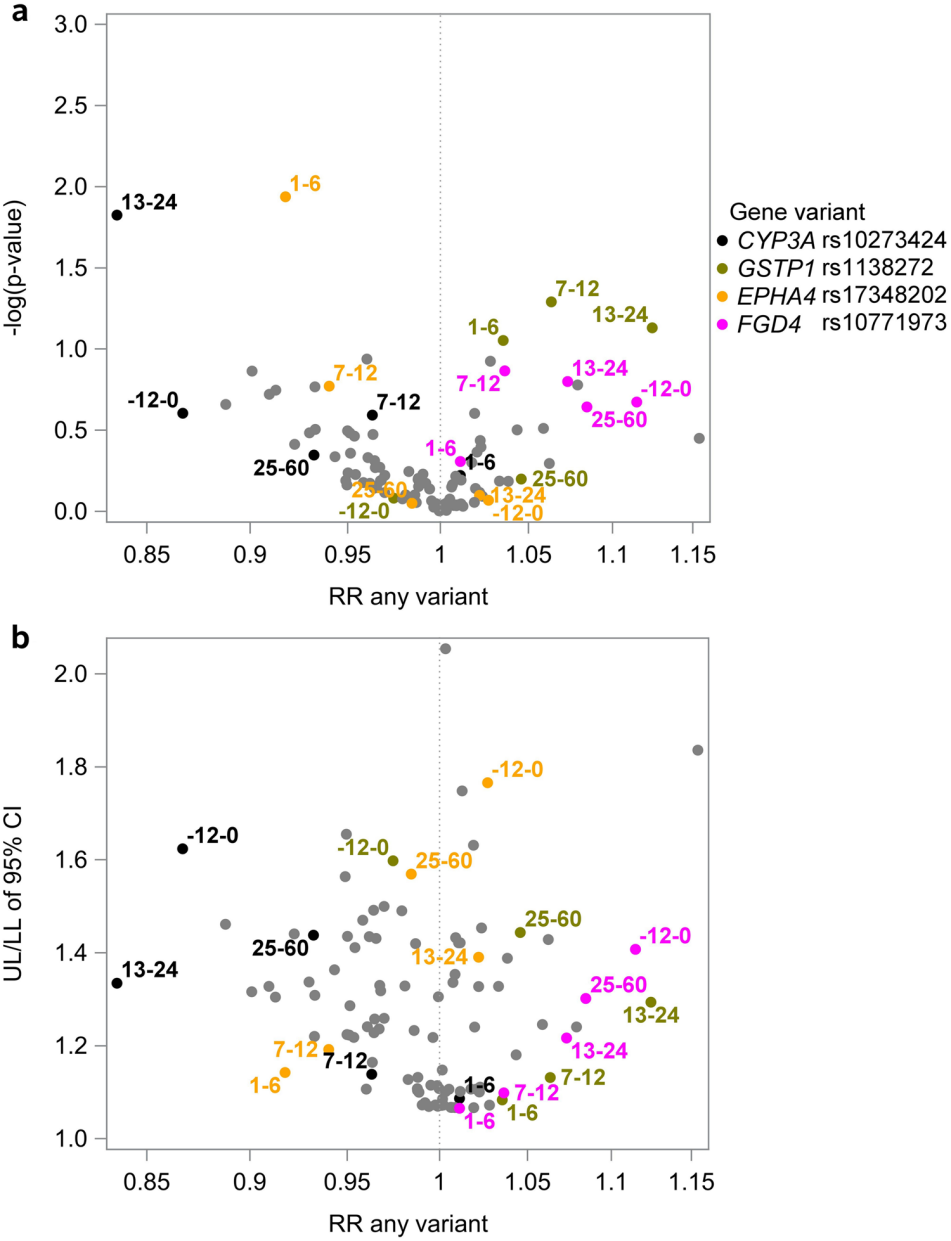
Associations between 21 SNPs and receipt of *health-related benefits* among 2430 premenopausal women with breast cancer in Denmark from 1 year before to 5 years after diagnosis (2007–2011). **a** Volcano plot: −  log(*p*) against the rate ratio. **b** Volcano plot: Ratio of the 95% CI upper and lower limit against the rate ratio. *CI* Confidence interval, *RR* rate ratio, *SNP* single nucleotide polymorphism, *UL/LL* the ratio between the upper and lower limit of the 95% confidence interval. Estimates are for variant carriers compared with non-carriers. Numbered labels refer to the time period in months since breast cancer diagnosis. We colored and labelled SNPs that stood out during at least one time period**.** A smaller UL/LL reflects a narrower confidence interval. Full results are available in Supplementary file2, Supplementary Table 4

**Fig. 3:**
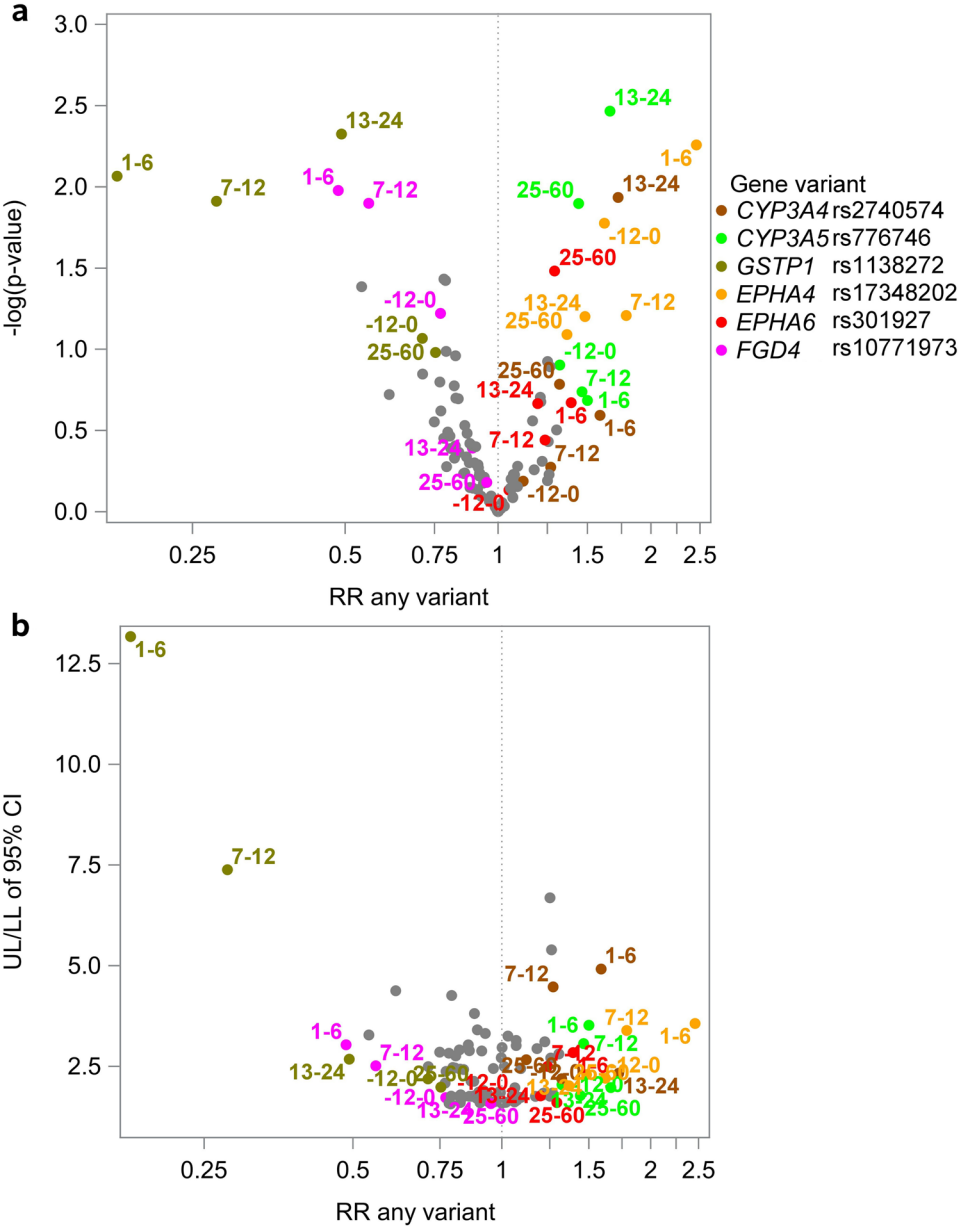
Associations between 21 SNPs and receipt of *labor market-related benefits* among 2430 premenopausal women with breast cancer in Denmark from 1 year before to 5 years after diagnosis (2007–2011). **a** Volcano plot: −  log(*p*) against the rate ratio. **b** Volcano plot: Ratio of the 95% CI upper and lower limit against the rate ratio. *CI* Confidence interval, *RR* rate ratio, *SNP* single nucleotide polymorphism, *UL/LL* the ratio between the upper and lower limit of the 95% confidence interval. Estimates are for variant carriers compared with non-carriers. Numbered labels refer to the time period in months since breast cancer diagnosis. We colored and labelled SNPs that stood out during at least one time period**.** A smaller UL/LL reflects a narrower confidence interval. NB: the scale on the y-axis of panel B differs from that of panel B in Figs. 2 and 4. Full results are available in Supplementary file2, Supplementary Table 5

**Fig. 4:**
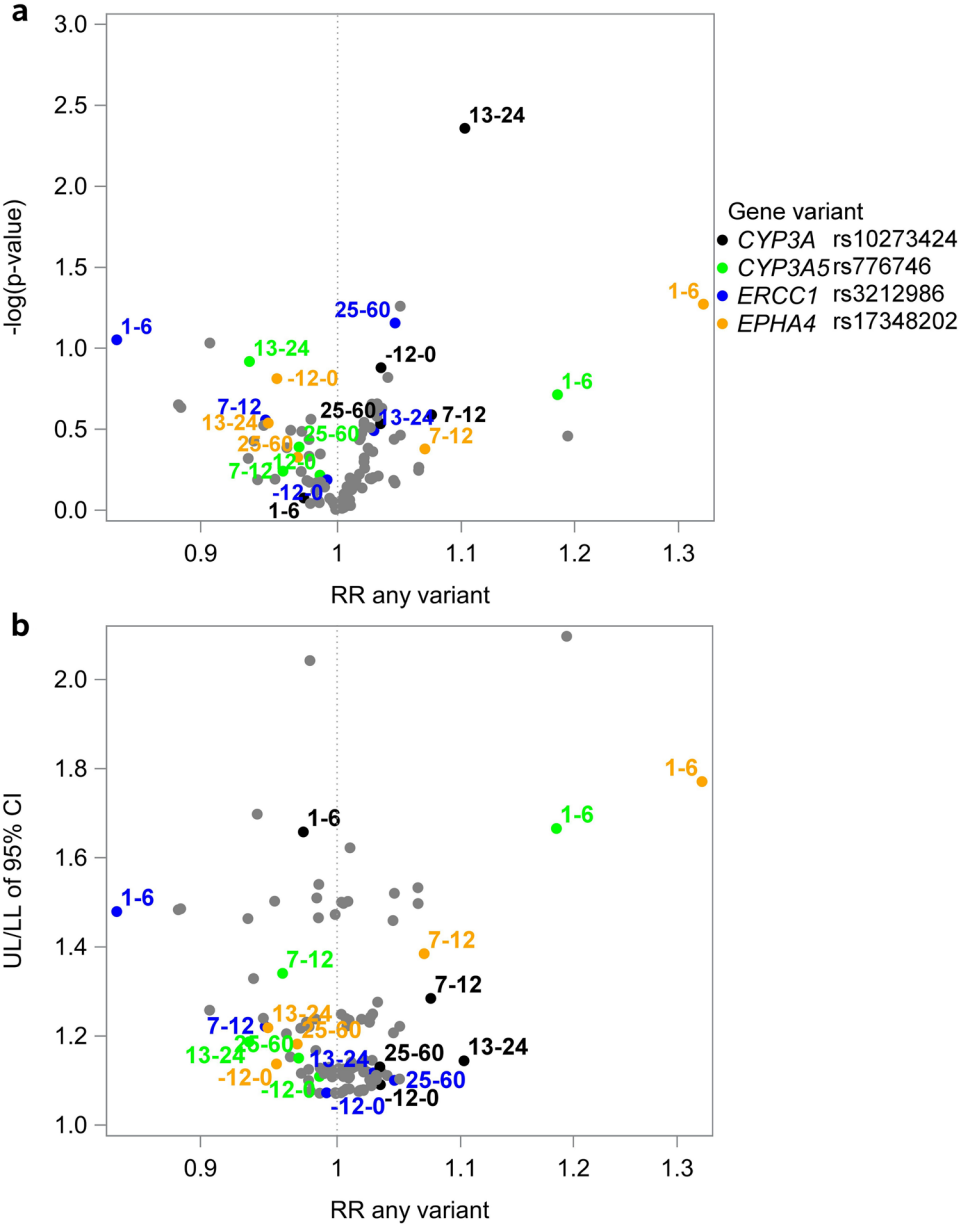
Associations between 21 SNPs and *self-support* among 2430 premenopausal women with breast cancer in Denmark from 1 year before to 5 years after diagnosis (2007–2011). **a** Volcano plot: − log(*p*) against the rate ratio**. b** Volcano plot: Ratio of the 95% CI upper and lower limit against the rate ratio. *CI* Confidence interval, *RR* rate ratio, *SNP* single nucleotide polymorphism, *UL/LL* the ratio between the upper and lower limit of the 95% confidence interval. Estimates are for variant carriers compared with non-carriers. Numbered labels refer to the time period in months since breast cancer diagnosis. We colored and labelled SNPs that stood out during at least one time period. A smaller UL/LL reflects a narrower confidence interval. Full results are available in ﻿Supplementary file2, Supplementary Table 6

Overall, we found no compelling evidence of associations between the examined SNPs and receipt of social benefits. However, associations between eight SNPs and receipt of social benefits stood out at one or more time periods for at least one of the social benefit groups (Figs. 2, 3 and 4), namely *CYP3A* rs10273424, *CYP3A4* rs2740574, *CYP3A5* rs776746, *GSTP1* rs1138272, *EPHA4* rs17348202, *EPHA6* rs301927, *ERCC1* rs3212986, and *FGD4* rs10771973.

#### Health-related benefits

Variant carriers of *GSTP1* rs1138272 (*n* = 365) were slightly more likely to receive health-related benefits than non-carriers from diagnosis until 24 months after diagnosis (RRs [95% CIs]: 1.04 [0.99, 1.08], 1.06 [1.00, 1.13], and 1.12 [0.99, 1.28] during 1–6, 7–12, and 13–24 months, respectively; Fig. 2; Supplementary file2, Supplementary Table 4). RRs and differences in the median number of weeks on health-related benefits were small. A similar pattern was observed for variant carriers of *FGD4* rs10771973 (*n* = 1169) from 7 to 60 months after diagnosis (RRs 1.04 [0.99, 1.09], 1.07 [0.97, 1.18], and 1.08 [0.95, 1.24] during 7–12, 13–24, and 25–60 months, respectively), but also in the year before diagnosis (RR: 1.11 [0.94, 1.32]).

Variant carriers of *CYP3A* rs10273424 (*n* = 398) received health-related benefits less often than non-carriers during 13–24 months after diagnosis (RR 0.84 [0.72, 0.97]; Fig. 2; Supplementary file2, Supplementary Table 4). The same applied to variant carriers of *EPHA4* rs17348202 (*n* = 231) in the six months directly following diagnosis (RR 0.92 [0.86, 0.98]). For *CYP3A* rs10273424, the median number of weeks of health-related benefits received during 13–24 months post-diagnosis was 3 (inter-quartile range [IQR]: 0, 26 weeks) for variant carriers and 6 (IQR 0, 35 weeks) for non-carriers. The corresponding number of weeks for *EPHA4* rs17348202 during 1–6 months were 26 (IQR 20, 26 weeks) and 26 (IQR 26, 26 weeks).

#### Labor market-related benefits

Receipt of labor market-related benefits was uncommon. The median number of weeks was 0 (IQR 0, 0 weeks) for all time periods in both carriers and non-carriers, except for variant carriers of *CYP3A5* rs776746 (*n* = 332) during 25–60 months post-diagnosis (0 [0, 5] weeks; Supplementary file2, Supplementary Table 5). While we observed relatively strong RR estimates between some SNPs and receipt of labor market-related benefits, confidence intervals were wide (Fig. 3; Supplementary file2, Supplementary Table 5).

#### Self-support

Carriers of *CYP3A* rs10273424 (*n* = 398) were more likely to be self-supporting 13–24 months after diagnosis than non-carriers (RR 1.10 [1.03, 1.18]; Fig. 4; ﻿Supplementary file2, Supplementary Table 6). The median number of weeks of self-support was 47 (IQR 12, 52 weeks) for variant carriers vs. 41 (IQR 2, 52 weeks) for non-carriers. Compared with non-carriers, during 1–6 months after diagnosis, we observed positive associations for self-support among variant carriers of *CYP3A5* rs776746 (*n* = 332; RR 1.18 [0.92, 1.53]) and *EPHA4* rs17348202 (*n* = 231; RR 1.33 [1.00, 1.76]), and an inverse association for *ERCC1* rs3212986 (*n* = 967; RR 0.84 [0.69, 1.03]). However, few women were self-supporting during this period, with a median of 0 (IQR: 0, 0) weeks for all three SNPs in both carriers and non-carriers.

#### Exploratory analyses

Results were not meaningfully changed for heterozygous or homozygous women (Supplementary file2, Supplementary Tables 4–6), nor in analyses restricted to women in the ER +/TAM + group (*n* = 1895; ﻿Supplementary file2, Supplementary Tables 7–9).

## Discussion

In this study of social benefit receipt from 1 year before through 5 years after breast cancer diagnosis, we found that approximately 80% of premenopausal breast cancer patients received health-related social benefits in the first six months after diagnosis. This percentage declined over the next six months, stabilizing at approximately 23% for the period 2–5 years after diagnosis, i.e., nearly a doubling in the proportion from the year before breast cancer diagnosis. These results are comparable with those of studies conducted in Sweden, which reported that 60% of breast cancer survivors were on sick leave shortly after diagnosis [7], and that 71% were on sick leave during the first year after diagnosis [34]. Proportions of breast cancer survivors on sick leave declined considerably over time, reaching 13%, 2 years after diagnosis [7] and 19%, 5 years after diagnosis [34]. Receipt of disability pension was relatively stable over time in the Swedish study, with 21–24% receiving a disability pension during the first to fifth year after diagnosis [34].

We found no evidence that the 21 candidate SNPs related to taxane metabolism, transport, toxicity, and DNA and neural repair were meaningfully associated with receipt of social benefits. Although associations for eight SNPs were suggested, estimates were close to null and/or imprecise due to low numbers. Variant carriers of *CYP3A* rs10273424 were at slightly lower risk of receiving health-related benefits and more likely to be self-supporting during the second year after diagnosis. Our previous findings for the ProBe CaRe cohort suggested that carriers compared with non-carriers of this SNP were more likely to return-to-work and to achieve stable attachment to the labor market within the first 2 years following breast cancer diagnosis [15]. However, in the same cohort, carriers of this variant had a higher risk of dying after a breast cancer diagnosis than non-carriers (hazard ratio [95% CIs]: 1.33 [0.98, 1.81]) [29]. The possible mechanisms underlying these findings are not clear. Although *CYP3A* rs10273424 is an intronic variant (i.e., non-coding), it could act via modified splicing or linkage disequilibrium with other SNPs [29]. Nevertheless, we cannot rule out chance. Furthermore, we observed that variant carriers of *GSTP1* rs1138272 and *FGD4* rs10771973 had marginally higher rates of health-related benefits than non-carriers after breast cancer diagnosis. Although this is in line with studies reporting associations between these SNPs and higher risk of taxane-induced peripheral neuropathy in variant carriers than non-carriers [10, 12], lack of replication is a pertinent issue [35, 36]. For *FGD4* rs10771973, we also observed an association in the year before diagnosis, and thereby before chemotherapy. Thus, differential toxicity of taxane-based chemotherapy between variant carriers and non-carriers is unlikely to explain our findings for this SNP. Larger studies are needed to robustly estimate the possible associations between the selected SNPs and receipt of social benefits, and the underlying mechanisms and any link to ability to work should be explored.

Given the lack of evidence of associations between the selected SNPs and social benefit receipt, other avenues for supporting women’s labor market attachment after breast cancer, should be considered. A recent analysis in our cohort found a social gradient in receipt of health-related benefits by level of education and cohabitation status [30]. Therefore, efforts to increase labor market attachment in survivors of breast cancer with short education and living alone may prove valuable.

The main strength of this study was its high-quality data and complete follow-up, limiting selection and information biases. The high-quality of the data stemmed from (i) using a population-based cohort identified from the DBCG database with high validity [26]; (ii) accessing detailed weekly registry-based data on social benefit payments from the DREAM database, which has previously been validated and regarded as suitable for health research [20, 21]; and (iii) applying extensive quality control to the SNP data [14, 15]. Only for *CYP1B1* rs1056836 were the observed and expected genotype frequencies meaningfully different (Supplementary file1, Supplementary Table 1).

Our study also had several limitations. First, we had no information on whether receiving health-related social benefits was related to taxane treatment or not. Furthermore, we used recommended chemotherapy rather than administered chemotherapy to avoid immortal time bias [37]. We also lacked information on dose, dose-reduction, discontinuation, and adverse effects of chemotherapy. Women experiencing side effects from chemotherapy, potentially due to genetic predisposition, might have received lower doses of chemotherapy, paused or discontinued their treatment. This could potentially lead to underestimation of the association between the selected SNPs and receipt of social benefits. In addition, docetaxel was given in combination with cyclophosphamide and for most patients, with epirubicin. Chemotherapy was followed by endocrine therapy for women with ER + tumors, and tamoxifen metabolism is also affected by genes of the CYP family [14]. For these reasons, we cannot necessarily assign any potential effect fully to docetaxel. Second, while we have accounted for time since breast cancer diagnosis, we have not accounted for changes in the general Danish economy, including unemployment rates and the legislation on social benefits. Such changes are, however, unlikely to impact our estimates of the associations between SNPs and social benefit receipt, assuming that the distributions of the SNPs are stable over time. Finally, we studied each SNP separately without considering their combined effect. Moreover, we genotyped DNA obtained from tumor tissue, which may not perfectly reflect the genotype influencing enzyme activity in the liver where docetaxel is metabolized. Nonetheless, earlier studies have reported excellent agreement between genotype for breast cancer patients ascertained in DNA from FFPE tumor tissue, and from both FFPE non-cancerous lymph node tissue [38, 39] and full blood leukocytes representing germline mutations [39].

In conclusion, we found no evidence of clinically meaningful associations between selected SNPs related to taxane metabolism, transport, toxicity, or DNA and neural repair, and receipt of social benefits among premenopausal breast cancer survivors.

## Supplementary Information

Below is the link to the electronic supplementary material.

Supplementary file1 (PDF 268 KB)

Supplementary file2 (XLSX 74 KB)

## Data Availability

The data used in our analyses for this paper cannot be shared, in accordance with Danish law. The data is not publicly accessible and is stored on servers at Statistics Denmark. The study protocol and statistical analysis plan are available upon request to the authors.

## Abbreviations

CCI: Charlson Comorbidity Index
CI: Confidence interval
DBCG: Danish Breast Cancer Group
DREAM: Danish Register for Evaluation of Marginalization
ER: Estrogen receptor
ER + /TAM +: Women with ER-positive tumors who received tamoxifen
ER −/TAM −: Women with ER-negative tumors who did not receive tamoxifen
FFPE: Formalin-fixed paraffin-embedded
HER2: Human epidermal growth factor receptor 2
IQR: Inter-quartile range
ProBe CaRe: Predictors of Breast Cancer Recurrence
RR: Rate ratio
rs: RefSeq
SNP: Single nucleotide polymorphisms
UL/LL: Ratio between the upper and lower limit of the 95% confidence interval
