# Supplementary material for: Genetic variants and social benefit receipt in premenopausal women with breast cancer treated with docetaxel: a Danish population-based cohort study
Source: Breast Cancer Res Treat. 2024 Sep 20;209(1):73–84. doi: 10.1007/s10549-024-07474-9 (PMC11785639; doi:10.1007/s10549-024-07474-9)
Supplement: Supplementary file 1 — Supplementary file1 (PDF 268 KB) [file 10549_2024_7474_MOESM1_ESM.pdf]

Online Resource 1: Supplementary Methods

Genetic variants and social benefit receipt in premenopausal women with breast cancer treated with docetaxel: a Danish population-based cohort study

Breast Cancer Research and Treatment

Julie A. Schmidt*, Cathrine F. Hjorth, Dóra K. Farkas, Per Damkier, Tore B. Stage, Søren Feddersen, Stephen Hamilton-Dutoit, Bent Ejlertsen, Timothy L. Lash, Thomas P. Ahern, Deirdre Cronin-Fenton

Content

Supplementary Figure 1. Flow chart of participants in the study. 2

Supplementary Table 1. Details of the 21 genetic variants available for the study 3

Supplementary Table 2. Codes from the Danish Register for Evaluation of Marginalization (DREAM) used to categorize social benefits.. 4

References 5

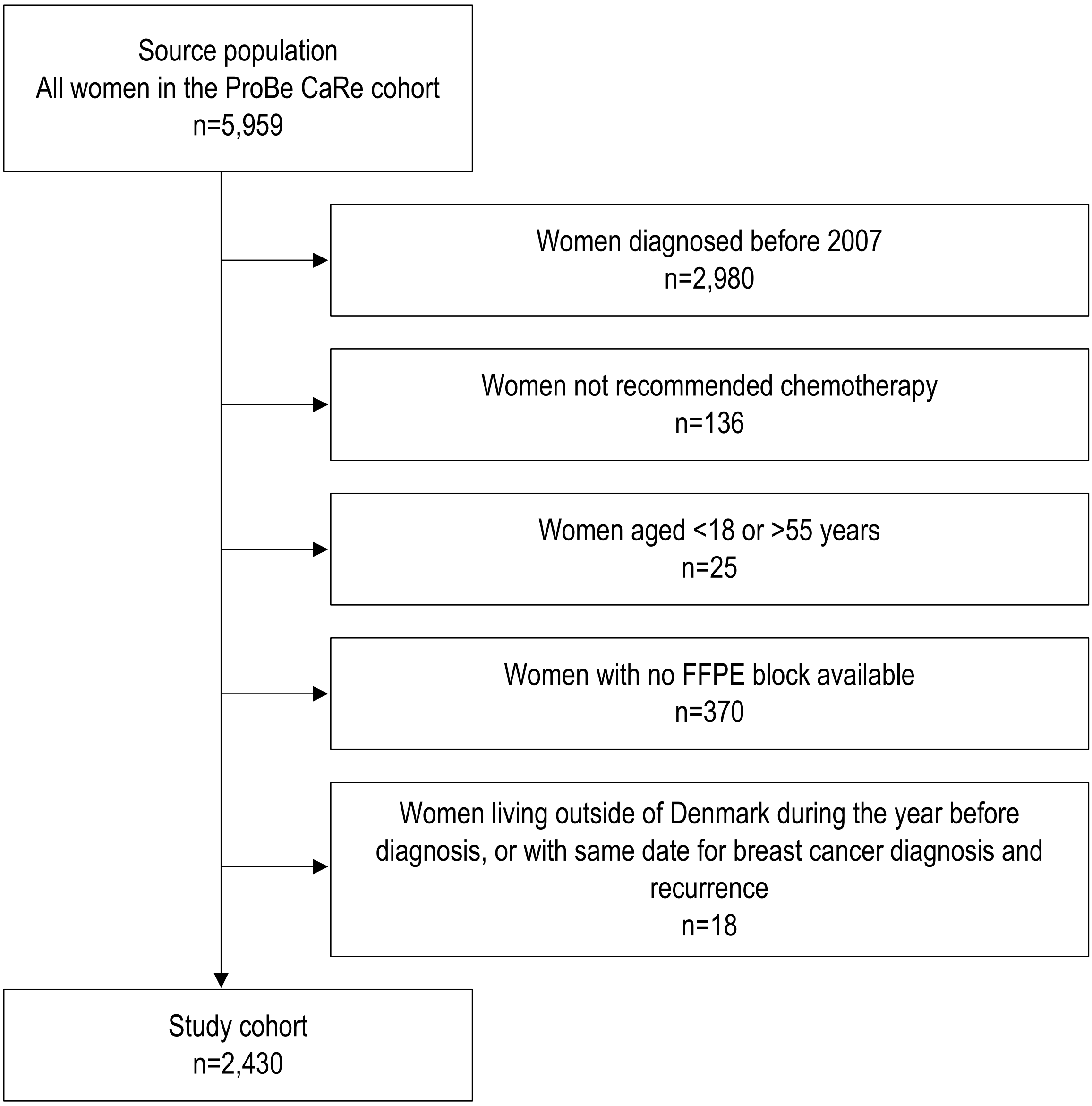

Supplementary Figure 1. Flow chart of participants in the study
Abbreviations: FFPE, Formalin-fixed paraffin-embedded; ProBe CaRe, Predictors of Breast Cancer Recurrence

Supplementary Table 1. Details of the 21 genetic variants available for the study

Gene	RefSeq number	Benchmark	TaqMan assay ID	Call rate	Minor allele	Frequency	Observed				Expected			HWE
		MAF[c]					Non-carriers	Hetero-zygote	Homo-zygote	N/A	Non-carriers	Hetero-zygote	Homo-zygote	Chi^2
Metabolism														
CYP1B1 [a]	rs1056836	43%	C___3099976_30	97%	C	44%	812	1093	508	72	461	1187	765	15.24
CYP3A [b]	rs10273424	9%	C__29554473_10	95%	A	8%	4076	748	31	232	4079	742	34	0.27
CYP3A4 [a]	rs2740574	4%	C___1837671_50	97%	G	4%	2231	177	12	64	2223	193	4	15.98
CYP3A4 [a]	rs35599367	5%	C__59013445_10	97%	T	3%	2251	144	8	82	2246	155	3	11.44
CYP3A5 [b]	rs776746	7%	C__26201809_30	100%	T	8%	4335	700	37	15	4328	715	30	2.22
GSTP1 [a]	rs1138272	9%	C___1049615_20	99%	T	8%	2090	352	19	24	2086	359	15	0.96
Transport														
ABCB1 [b]	rs1045642	47%	C___7586657_20	98%	G	45%	1508	2526	966	87	1536	2471	994	2.51
ABCB1 [b]	rs1128503	44%	C___7586662_10	97%	A	43%	1652	2371	930	134	1626	2424	904	2.36
ABCB1 [b]	rs2032582	46%	C_11711720C_30	96%	A	45%	1485	2373	1011	218	1466	2411	992	1.24
ABCC2 [a]	rs12762549	48%	C__11214917_10	97%	G	46%	733	1138	544	69	702	1200	513	6.47
ABCG2 [a]	rs2231142	11%	C__15854163_70	98%	A	10%	1961	446	25	53	1961	445	25	0.00
SLCO1B1 [a]	rs2306283	40%	C_1901697_20	96%	C	41%	860	1095	432	98	830	1155	402	6.47
SLCO1B1 [a]	rs4149056	16%	C__30633906_10	96%	C	14%	1771	570	55	89	1764	584	48	1.28
SLCO1B3 [a]	rs11045585	14%	C__31106434_10	98%	G	14%	1826	559	59	51	1814	583	47	4.22
DNA repair														
ERCC1 [a]	rs11615	37%	C___2532959_1_	99%	G	37%	1118	991	341	35	1139	981	330	0.82
ERCC1 [a]	rs3212986	25%	C_2532948_10	98%	A	23%	1441	869	121	54	1447	857	127	0.47
ERCC2 [a]	rs13181	38%	C___3145033_10	97%	G	37%	1008	1025	371	81	962	1118	325	16.51
Toxicity and neural repair														
EPHA4 [a]	rs17348202	6%	C__34414779_10	98%	C	5%	2205	223	12	45	2199	234	6	1.28
EPHA5 [a]	rs7349683	35%	C___1336545_30	99%	T	35%	1025	1116	307	37	1024	1118	305	0.01
EPHA6 [a]	rs301927	16%	C___1037994_10	98%	G	17%	1665	673	86	61	1653	698	74	3.04
FGD4 [a]	rs10771973	30%	C__30728517_30	99%	A	29%	1249	991	208	37	1243	1003	202	0.33

Table adapted from Hjorth *et al.* [1]
[a] Genotyped as part of studies by Hjorth *et al.* [1, 2]
[b] Genotyped as part of Ahern *et al.* [3]
[c] Minor allele frequencies in the non-Finnish European female population.
Abbreviations: HWE, Hardy-Weinberg Equilibrium; MAF, minor allele frequency; N/A, not available.

Supplementary Table 2. Codes from the Danish Register for Evaluation of Marginalization (DREAM) used to categorize social benefits

Category	Codes	Description
**Self-supporting**	Empty cell	Considered employed
	521	Trainee, adult
	413	Leave-of-absence due to education
	651, 652, 661, 662, 794	State Education Fund grants
	122, 123	Vacation payment from employment
	996	Pension age without pension payment
**Health-related benefits**	771, 774, 895, 761,762, 769,772, 773,779, 782,796	Flexi job for those with a reduced workability
	774, 890, 893-899, 891, 892	Sick leave benefit[a]
	740, 743-748	Unemployed awaiting flexi job
	750, 753-758, 760, 763-768, 791, 792	Rehabilitation
	784, 810, 813-819	Vocational rehabilitation program
	785, 870, 873-879	Workability clarification
	781, 783, 793, 797	Disability pension
	622	Early retirement after flexible job
**Labor market-related benefits**	112, 113, 115	Unemployment benefit part time
	411	Leave-of-absence schemes
	111	Unemployment benefit all week
	114	Unemployed without benefit
	121, 124-126	Vacation payment from unemployment
	130-139, 141, 142, 152, 153, 730-739, 741	Social assistance, not health related
	160, 163-169	Ready for employment benefit for immigrants
	710-719	Social benefit, immigrants
	704-709	Immigration benefit during special efforts e.g., job training
	140-149, 151, 414, 700, 703, 720-729, 732, 742, 751, 752	Education assistance, not health related
	211-219, 221, 222, 224, 225, 231, 232, 297-299, 511, 522, 541, 722, 759	Unemployment benefit during special efforts e.g., job training or supervision

During weeks of maternity leave (codes 412 and 881), we categorized women according to the code in the week immediately before the start of their maternity leave. For example, if a woman received sick leave benefits the week before starting maternity leave, we categorized them as receiving health-related benefits throughout the maternity leave.
Codes 611, 621 (early retirement), 997 (emigration), 998 (retirement), and 999 (death) were used for censoring.
[a] ‘Short-term’ sick leave is paid by the employer and thus not captured by DREAM; during the study period, “short-term” ranged from 14 to 30 days [4].

References

1. Hjorth CF, Damkier P, Stage TB, Feddersen S, Hamilton-Dutoit S, Rørth M, Ejlertsen B, Lash TL, Ahern TP, Sørensen HT, Cronin-Fenton D (2022) Single-nucleotide polymorphisms and the effectiveness of taxane-based chemotherapy in premenopausal breast cancer: a population-based cohort study in Denmark. Breast Cancer Res Treat 194:353-363. DOI: 10.1007/s10549-022-06596-2
2. Hjorth CF, Damkier P, Stage TB, Feddersen S, Hamilton-Dutoit S, Ejlertsen B, Lash TL, Bøggild H, Sørensen HT, Cronin-Fenton D (2023) The impact of single nucleotide polymorphisms on return-to-work after taxane-based chemotherapy in breast cancer. Cancer Chemother Pharmacol 91:157-165. DOI: 10.1007/s00280-022-04499-z
3. Ahern TP, Collin LJ, Baurley JW, Kjærsgaard A, Nash R, Maliniak ML, Damkier P, Zwick ME, Isett RB, Christiansen PM, Ejlertsen B, Lauridsen KL, Christensen KB, Silliman RA, Sørensen HT, Tramm T, Hamilton-Dutoit S, Lash TL, Cronin-Fenton D (2020) Metabolic Pathway Analysis and Effectiveness of Tamoxifen in Danish Breast Cancer Patients. Cancer Epidemiol Biomarkers Prev 29:582-590. DOI: 10.1158/1055-9965.Epi-19-0833
4. DA Analysis (2019) Store omkostninger ved sygefravær gør det svært for virksomhederne at integrere folk på kanten af arbejdsmarkedet [Extensive costs of sick leave make it difficult for companies to integrate people on the edge of the labour market]. Available from: https://www.da.dk/politik-og-analyser/beskaeftigelse/2019/store-omkostninger-ved-sygefravaer-goer-det-svaert-for-virksomhederne-at-integrere-folk-paa-kanten-af-arbejdsmarkedet/. Accessed 6 Mar 2024
